# False acetabulum is preoperative guidance for Crowe type IV hips on hip reduction without femoral shortening during total hip arthroplasty

**DOI:** 10.1111/ans.17119

**Published:** 2021-08-10

**Authors:** Jing‐Yang Sun, Bo‐Han Zhang, Jun‐Min Shen, Yin‐Qiao Du, Yong‐Gang Zhou

**Affiliations:** ^1^ Medical School of Chinese PLA Beijing China; ^2^ Department of Orthopedics, the First Medical Center Chinese PLA General Hospital Beijing China; ^3^ Medical School of Nankai University Tianjin China

**Keywords:** developmental dysplasia, false acetabulum, femoral shortening, high dislocated hips, total hip arthroplasty

## Abstract

**Background:**

We aimed to analyze if the false acetabulum is a good indicator for determining femoral shortening.

**Methods:**

We retrospectively included 102 patients with unilateral Crowe type IV developmental dysplasia who underwent primary total hip arthroplasty from April 2008 to May 2019. Based on the presence of false acetabulum, the 102 hips were further classified Crowe IVA group and Crowe IVB group. Radiographic measurement included the height of greater trochanter (HGT) preoperatively and postoperatively, which reflected the distalisation of greater trochanter (DGT). Harris hip score (HHS), limb length discrepancy (LLD), and complications were collected as clinical evaluation.

**Results:**

Sixty hips were classified into Crowe IVA group, and 42 hips were classified into Crowe IVB group. Within Crowe IVA group, the proportion of hips treated with subtrochanteric osteotomy was significantly higher than that in Crowe IVB group (97% vs. 12%) (*P* < 0.001). The DGT in Crowe IVA group was also greater (67 vs. 32 mm) (*P* < 0.001). At last follow‐up, both two groups obtained excellent clinical scores. There was no significant difference in postoperative LLD between the two groups (*P* = 0.001). Six dislocations occurred and three patients developed femoral nerve palsy, while all recovered in a year.

**Conclusion:**

The false acetabulum is a promising and good indicator for determining femoral shortening.

## Graphical abstract

The false acetabulum is a promising and good indicator for determining femoral shortening in high dislocated hips. Although treated with different procedures, similarly excellent clinical outcomes have been obtained in Crowe type IV hips with or without false acetabulum.

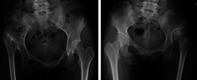

## Introduction

Total hip arthroplasty (THA) in developmental dysplasia of the hip (DDH) is a technically demanding procedure due to morphological variations.^1^, ^2^, ^3^ In the setting of severe dysplasia (Crowe type IV or Hartofilakidis type C),^2^, ^3^ high riding hip centers and soft‐tissue contractures are quite likely to be encountered by surgeons.^4^ Therefore, femoral shortening may be necessary for anatomic placement of the acetabular component to avoid neurovascular complications.

Over the years, several methods emerged aiming for the avoidance of femoral shortening, including intraoperative injection of muscle relaxant, more extensive soft tissue release, leverage and progressive femoral lowering by iliofemoral distraction.^5^, ^6^, ^7^ THA without femoral shortening was possible in Crowe type IV hips, which also provided good clinical outcomes and acceptable complication rates.^7^, ^8^ In some studies, femoral shortening was recommended when the lower limb would be lengthened more than 3–5 cm in dislocated hips to decrease the risk of excessively stretching the sciatic nerve.^9^, ^10^, ^11^ Besides, multimodal intraoperative monitoring has been described to detect early warning signs of nerve injury and offer the guidance.^10^ Interestingly, the radiographic measurements of Xu et al.^11^ and Ma et al.^12^ confirmed that proximal femur in high dislocated hips without false acetabulum was narrower, with little metaphysis flare to mate with the stem, and often migrated more superiorly than that in hips with false acetabulum formation. They presumed that the limb of the former type might gain more lengthening when repositioning the hip into the anatomical center of rotation; thus, subtrochanteric osteotomy was more likely to be performed.

Considering the potential relationship between the false acetabulum and femoral anatomical morphology, we hypothesized that hip reduction can be achieved without femoral shortening in Crowe type IV DDH with the presence of false acetabulum. The purpose of this study is to investigate if the false acetabulum is a good indicator for determining femoral shortening.

## Methods

### Patients

After Institutional Review Board approval, we retrospectively reviewed 171 consecutive patients affected by unilateral Crowe type IV DDH who underwent primary THA with modular cementless stem (S‐ROM, DePuy, Warsaw, Indiana) from April 2008 to May 2019 in our institution. The mean follow‐up time was 70 months (from 19 to 132 months).

Exclusion criteria were (1) previous femoral osteotomy, or (2) previous hip pyogenic arthritis, or (3) proximal placement of the cup component at high hip center, which meant the rotation center was at least 22 mm above the inter‐teardrop^13^ or (4) use of conical sleeve, or (5) inadequate postoperative anteroposterior (AP) radiographs. Finally, a total of 102 patients were included in the study. Based on the presence of false acetabulum, the 102 hips were further classified Crowe IVA group and Crowe IVB group. The Crowe IVA group had completely dislocation without false acetabulum. The Crowe IVB group had completely dislocated with false acetabulum formation.

**Fig 1:**
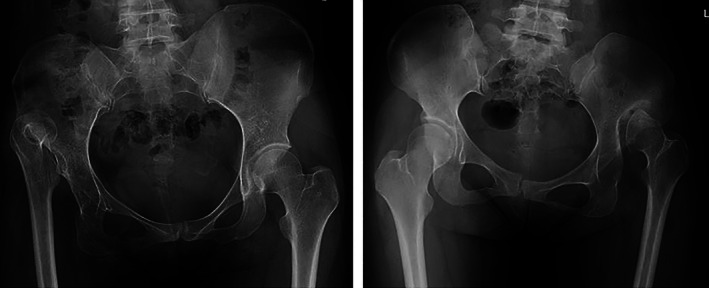
(a) Absence of false acetabulum, there was no sign of osteosclerosis; (b) false acetabulum formation, the femoral head reached the lateral margin of ilium wing, accompanied with the formation of high‐density osteosclerotic zone and osteophyte.

The presence of false acetabulum was evaluated by the AP radiographs. When the femoral head is articulated with a false acetabulum, it often reached the lateral margin of the ilium wing, accompanied with the formation of a high‐density osteosclerotic zone and osteophyte. Otherwise, the absence of an area of osteosclerosis proximal to the true acetabulum suggested there was no false acetabulum (Fig. 1).

### Radiographic assessment

Standardized digital, calibrated AP hip and full‐length standing AP radiographs were acquired both preoperatively and postoperatively. The performance of subtrochanteric osteotomy was validated by osteotomy line on images of the second day postoperatively and reference to operative notes. Radiographic measurement included the measuring of the height of greater trochanter (HGT) preoperatively and postoperatively, which can be used to calculate the distalisation of greater trochanter (DGT).^14^ The HGT was the vertical distance from the most proximal point of the greater trochanter to the inter‐teardrop line. The DGT was the value of the difference of HGT before and after the surgery, which indirectly reflected the change of leg length without femoral shortening and the necessity of femoral shortening，especially among the patients in the Crowe IVA group.

### Surgical procedure

All operations were performed by one senior surgeon using the posterolateral approach under general anesthesia in the lateral decubitus position. The joint capsule was totally excised. Tensor fasciae latae, gluteal sling release, and iliopsoas tendon was conventionally released. In order to use ceramic on ceramic bearing, the cup (range 44–46 mm) was implanted at the anatomic position by reaming the acetabulum posteriorly and inferiorly.^15^ After the cup implantation, the femoral canal was prepared using the reamer for the S‐ROM stem. With trial seated in the femur, we measured the final vertical distance from the femoral head to cup under constant and vigorous traction. If hip reduction with a femoral trial stem was impossible, a subtrochanteric osteotomy would be performed.

The osteotomy position was planned to be adjacent enough to the end of the proximal sleeve, approximately corresponding to 1–2 cm beneath the lesser trochanter. Prophylactic cerclage wires were placed both proximally and distally around the fragment of the planned osteotomy. Also, a longitudinal line along the femoral diaphysis was marked by electrocautery prior to osteotomy to determine the rotation. After removing the trial, a transverse osteotomy was performed, by resection of a length of the femur below the lesser trochanter. The length of the removed bone stock was based on the distance we measured before, leaving a scope of 1–1.5 cm with the surgeon's discretion. After completion of osteotomy, a final preparation of the femur was undertaken until optimal cortical contact was achieved. Then, the trial reduction was performed again. If impossible, additional bone was incrementally resected at the osteotomy site until reduction was achieved. After trial reduction, stability, limb length and soft tissue tension were evaluated. Intraoperative measurement of limb length discrepancy (LLD) was performed by palpating the inferior point of the bilateral patella. Mild LLD could be adjusted by means of the modifications of head/neck length and stem depth in femur. Finally, the definitive femoral component was implanted with the rotational alignment of the femoral stem adjusted to allow approximately 30°–50° of combined anteversion. No rotational adjustment of the bone segment was performed. At last, motion in the abduction was assessed to evaluate the necessity of a percutaneous partial adductor tenotomy. Postoperatively, patient's hip and knee were maintained in flexion for several days to relax the sciatic nerve and reduce the tension of soft tissue.

Patients were followed up in regular intervals at 3.6 months, and yearly after surgery. Clinical information was collected including Harris hip score (HHS), LLD (measured from anterosuperior iliac spine to the prominence of medial malleolus), and occurrence of complications.

### Statistical analysis

All statistical analyses were performed using SPSS 26.0 (IBM Inc., Armonk, New York). Categorical variables were presented as frequencies and continuous variables as means ± SD. Continuous variables were assessed using Student's *t*‐test, whereas categorical variables were analyzed using chi‐squared test or Fisher's exact test. *P*‐value of <0.05 was considered significant.

## Results

In this study, 60 hips were classified into Crowe IVA group, and 42 hips were classified into Crowe IVB group. Demographic data were summarized in Table 1. Within Crowe IVA group, the proportion of hips treated with subtrochanteric osteotomy was significantly higher than that in Crowe IVB group (*P* < 0.001).

**Table 1:** Patient characteristics

	Total (*n* = 102)	Crowe IVA (*n* = 60)	Crowe IVB (*n* = 42)	*P*‐value
Age (years)	38.31 ± 11.48	39.67 ± 12.02	36.38 ± 10.50	0.156
Height (m)	1.59 ± 0.07	1.59 ± 0.06	1.60 ± 0.08	0.464
Weight (kg)	56.98 ± 10.47	56.99 ± 10.31	56.98 ± 10.81	0.996
BMI (kg/m2)	22.26 ± 3.37	22.38 ± 3.36	22.08 ± 3.43	0.664
Gender				
Male	9 (9%)	4 (7%)	5 (12%)	0.573
Female	93 (91%)	56 (93%)	37 (88%)	
Femoral shortening				
With STO	63 (62%)	58 (97%)	5 (12%)	<0.001
Without STO	39 (38%)	2 (3%)	37 (88%)	
Follow‐up (months)	45.14 ± 25.41	47.10 ± 26.01	42.33 ± 24.56	0.354

Abbreviations: BMI, body mass index; STO, subtrochanteric osteotomy.

**Fig 2:**
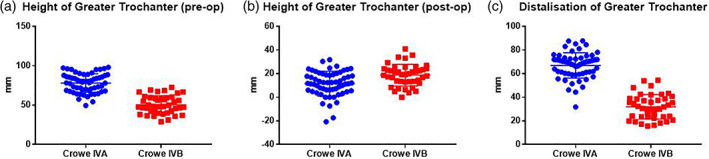
Comparison of height of greater trochanter (HGT) preoperatively and postoperatively along with distalisation of greater trochanter (DGT) between Crowe IVA and Crowe IVB group.

Before operation, the mean HGT was evidently greater in hips without false acetabulum (*P* < 0.001). Instead, the mean HGT postoperatively was significantly smaller in hips without false acetabulum (*P* < 0.001). Thus, the calculated value of DGT in Crowe IVA group was significantly greater than that in Crowe IVB group (67 vs. 32 mm) (*P* < 0.001) (Fig. 2). Among the five hips undergoing subtrochanteric osteotomy in Crowe IVB group, the mean DGT was 47 mm (range, 34–54 mm). Besides, the DGT was 32 and 46 mm, respectively, in hips without femoral shortening.

At the last follow‐up, both two groups obtained excellent clinical scores (HHS). Regarding the postoperative LLD, there was a significant difference between two groups (*P* = 0.001), which showed a tendency of shortening in Crowe IVA group of which hips mainly underwent subtrochanteric osteotomy (Table 2).

**Table 2:** Limb length discrepancy (LLD) and clinical outcomes (Harris hip score, HHS)

	Crowe IVA group (*n* = 60)	Crowe IVB group (*n* = 42)	*P*‐value
Postoperative LLD (cm)	−0.66 ± 0.84 (−2.5 ~ 0.85)	0.13 ± 0.73 (−1.8 ~ 1.4)	0.001
Preoperative HHS (points)	36.72 ± 8.92	37.02 ± 10.66	0.875
Postoperative HHS (points)	87.65 ± 6.65	88.69 ± 8.19	0.481
*P*‐value	0.000	0.000	

Totally six dislocations (at 1, 2, and 3 months) occurred in six patients (five in Crowe IVA group with femoral shortening and one in Crowe IVB group without femoral shortening). Four hips were treated by closed reduction and two by open reduction. Three patients (all in Crowe IVA group with femoral shortening) developed femoral nerve palsy with skin numbness on the frontal thigh or tibia and all recovered in a year. And there were no other complications such as sciatic nerve injury, fracture, non‐union or infection.

## Discussion

Due to morphological variations of high dislocated hips, it is generally difficult for reduction especially when the cup is reconstructed at the anatomical position.^4^ Current studies mainly focused on the outcomes of THA with or without femoral shortening.^5^, ^7^, ^8^, ^16^ Few studies investigated the indicator guiding the use of femoral shortening. In this study, we found that hip reduction without femoral shortening is very likely for Crowe type IV hips with the presence of false acetabulum.

As was described in Hartofilakidis^3^ classification systems, high dislocated hips were subdivided into two different types depending on the presence of false acetabulum. With the aid of those subtypes, they expected to predict the acetabular bone deficiencies encountered during THA and plan for reconstruction. Afterwards, Xu et al.^11^ and Ma et al.^12^ measured the canal flare index and dislocation height of femur in high dislocated hips on AP radiographs. Both of their results showed in hips without false acetabulum, more amount of dislocation height appeared in theproximal femur. It was speculated that the morphological difference between the two subtypes could be attributed to the stress stimulus provided by false acetabulum according to Wolf's law. In general, we supposed that the lower dislocation height and more insertion depth of stem in hips of Crowe IVB group can give less contribution to the limb lengthening. This is also validated by the low rate of applying subtrochanteric osteotomy in Crowe IVB group. More studies like computerized three‐dimensional planning may be needed to recognize the effect of false acetabulum on femoral morphology.

Our results of DGT indirectly identify the role of false acetabulum for avoiding femoral shortening. The same tendency was observed by Nagoya et al.^14^ and Fujishiro et al.^17^ Nagoya et al. included 20 hips with Crowe type IV of which eight were previously treated with femoral osteotomy. Their results showed the mean DGT was 36.3 mm in patients with iliofemoral osteoarthritis (OA), and 67.8 mm in patients without iliofemoral OA. Fujishiro et al. conducted a similar study with a larger scale (70 hips) and the mean DGT of hips with or without iliofemoral OA were 49.3 and 71.5 mm. However, it is important to note that iliofemoral OA described in their essays does not equate to the presence of false acetabulum. From the perspective of morphological development, we think it is not the pseudarthrosis between the pelvic wall and the femoral head resulting from long‐term abrasion, but a fossa forming at an early age providing load stimulus and obstructing higher dislocation that counts.

Although false acetabulum was a potent indicator, there was still a need of femoral shortening in five hips (12%) of Crowe IVB group. We speculated relatively higher dislocation in those cases might explain it. It must be admitted that the decision on femoral shortening can be influenced by many factors. Apart from limb length change, others like previous femoral osteotomy, history of pyogenic arthritis, inelastic scars due to multiple surgeries and rigid spine/pelvis deformity should be considered preoperatively. In this study, although treated with different procedures, most of hips within both two groups obtained excellent clinical scores and acceptable limb length equalization. It was reported that the incidence of sciatic nerve injury reached up to 5.2% in THA performed for DDH,^18^ because the sciatic nerve could deviate from its normal position due to hip dislocation or muscle contracture and the complexity of surgery.^18^, ^19^ In addition, excessive tension resulting from limb lengthening might be the main cause of sciatic nerve palsy.^13^, ^18^, ^19^ Fortunately, there is no such complication in our study, except for a slight femoral nerve palsy.

There are several limitations. First, this was a retrospective study. Fortunately, we mainly relied on the radiographs with less effect from recall bias. Second, several factors such as history of femoral osteotomy or pyogenic arthritis were excluded, aiming to determine the effect of false acetabulum by itself. Therefore, a comprehensive, multivariate analysis may be needed. Finally, it was a single‐institution, single‐surgeon study with use of a single femoral prosthesis, and has limited external validity. However, to our knowledge, it has reported the largest sample of unilateral Crowe type IV hips so far. We believe that subtypes based on the presence of false acetabulum can indeed remind surgeons of potential different interventions on femoral side in Crowe type IV hips.

## Conclusions

The false acetabulum is a promising and good indicator for determining femoral shortening. Although treated with different procedures, similarly excellent clinical outcomes have been obtained in both Crowe IVA and Crowe IVB group. However, there are some other factors influencing hip reduction. A multivariate analysis and morphological study are required to further validate the role of the false acetabulum.

## Author contributions

**Jing‐yang Sun:** Conceptualization; formal analysis; investigation; methodology; validation; writing‐original draft. **Bo‐han Zhang:** Conceptualization; formal analysis; investigation; methodology; resources; writing‐original draft. **Jun‐min Shen:** Resources; software. **Yin‐qiao Du:** Data curation; software. **Yonggang Zhou:** Conceptualization; formal analysis; supervision; validation; writing‐review & editing.

## Conflicts of interest

None declared.

## Ethical approval

This retrospective review study involving human participants was in accordance with the ethical standards of the institutional and national research committee and with the 1964 Helsinki Declaration and its later amendments or comparable ethical standards. This study was approved by the medical ethics committee of our hospital.

## Data Availability

All the data is from the Yonggang Zhou, ygzhou301@163.com.

## References

[1] Kosuge D , Yamada N , Azegami S , Achan P , Ramachandran M . Management of developmental dysplasia of the hip in young adults: current concepts. Bone Joint J. 2013;95‐B:732–7. 10.1302/0301-620X.95B6.31286 23723265

[2] Crowe JF , Mani VJ , Ranawat CS . Total hip replacement in congenital dislocation and dysplasia of the hip. J Bone Joint Surg Am. 1979;61:15–23. 365863

[3] Hartofilakidis G , Yiannakopoulos CK , Babis GC . The morphologic variations of low and high hip dislocation. Clin Orthop Relat Res. 2008;466:820–4. 18288552 10.1007/s11999-008-0131-9 PMC2504667

[4] Bicanic G , Barbaric K , Bohacek I , Aljinovic A , Delimar D . Current concept in dysplastic hip arthroplasty: techniques for acetabular and femoral reconstruction. World J Orthop. 2014;5:412–24. 25232518 10.5312/wjo.v5.i4.412 PMC4133448

[5] Li H , Yuan Y , Xu J , Chang Y , Dai K , Zhu Z . Direct leverage for reducing the femoral head in total hip arthroplasty without femoral shortening osteotomy for Crowe type 3 to 4 dysplasia of the hip. J Arthroplasty. 2018;33:794–9. 29269273 10.1016/j.arth.2017.09.011

[6] Lai KA , Liu J , Liu TK . Use of iliofemoral distraction in reducing high congenital dislocation of the hip before total hip arthroplasty. J Arthroplasty. 1996;11:588–93. 8872580 10.1016/s0883-5403(96)80114-8

[7] Imbuldeniya AM , Walter WL , Zicat BA , Walter WK . Cementless total hip replacement without femoral osteotomy in patients with severe developmental dysplasia of the hip: minimum 15‐year clinical and radiological results. Bone Joint J. 2014;98‐B:1449–54. 10.1302/0301-620X.96B11.33698 25371455

[8] Li H , Xu J , Qu X , Mao Y , Dai K , Zhu Z . Comparison of total hip arthroplasty with and without femoral shortening osteotomy for unilateral mild to moderate high hip dislocation. J Arthroplasty. 2017;32:849–56. 27919583 10.1016/j.arth.2016.08.021

[9] Kerboull M , Hamadouche M , Kerboull L . Total hip arthroplasty for Crowe type IV developmental hip dysplasia: a long‐term follow‐up study. J Arthroplasty. 2001;16:170–6. 11742471 10.1054/arth.2001.28368

[10] Kong X , Chai W , Chen J , Yan C , Shi L , Wang Y . Intraoperative monitoring of the femoral and sciatic nerves in total hip arthroplasty with high‐riding developmental dysplasia. Bone Joint J. 2019;101‐B:1438–46. 10.1302/0301-620X.101B11.BJJ-2019-0341.R2 31674243

[11] Xu H , Zhou Y , Liu Q , Tang Q , Yin J . Femoral morphologic differences in subtypes of high developmental dislocation of the hip. Clin Orthop Relat Res. 2010;468:3371–6. 20480403 10.1007/s11999-010-1386-5 PMC2974896

[12] Ma HY , Zhou YG , Zheng C , et al. New classification of Crowe type IV developmental dysplasia of the hip. Zhongguo Gu Shang. 2016;29:119–24. 27141778

[13] Fukui K , Kaneuji A , Sugimori T , et al. How far above the true anatomic position can the acetabular cup be placed in total hip arthroplasty? J Hip Int. 2013;23:129–34. 10.5301/hipint.5000010 23543468

[14] Nagoya S , Kaya M , Sasaki M , Tateda K , Kosukegawa I , Yamashita T . Cementless total hip replacement with subtrochanteric femoral shortening for severe developmental dysplasia of the hip. J Bone Joint Surg Br. 2009;91:1142–7. 19721037 10.1302/0301-620X.91B9.21736

[15] Zhou Y , Sun C , Wang Y . New method addressing the problem of using ceramic‐on‐ceramic bearing in too small acetabulum of high‐riding DDH patients with THA. Semin Arthro. 2012;23:226–31.

[16] Ollivier M , Abdel MP , Krych AJ , Trousdale RT , Berry DJ . Long‐term results of Total hip arthroplasty with shortening subtrochanteric osteotomy in Crowe IV developmental dysplasia. J Arthroplasty. 2016;31:1756–60. 26952206 10.1016/j.arth.2016.01.049

[17] Fujishiro T , Nishiyama T , Hayashi S , Kurosaka M , Kanno T , Masuda T . Leg length change in total hip arthroplasty with subtrochanteric femoral shortening osteotomy for Crowe type IV developmental hip dysplasia. J Arthroplasty. 2012;27:1019–22. 22480527 10.1016/j.arth.2012.01.032

[18] Lai KA , Shen WJ , Huang LW , et al. Cementless total hip arthroplasty and limb‐length equalization in patients with unilateral Crowe type‐IV hip dislocation. J Bone Joint Surg Br. 2005;87:339–45. 10.2106/JBJS.D.02097 15687157

[19] Eggli S , Hankemayer S , Müller ME . Nerve palsy after leg lengthening in total replacement arthroplasty for developmental dysplasia of the hip. J Bone Joint Surg Br. 1999;81:843–5. 10530847 10.1302/0301-620x.81b5.9610

